# Clozapine-Induced Dysarthria in a Patient With Treatment-Resistant Schizophrenia

**DOI:** 10.1192/bjo.2026.11888

**Published:** 2026-06-30

**Authors:** Juliet Raphael, Harishnath Ramachandran, Riya Basu

**Affiliations:** Birmingham and Solihull Mental Health Foundation Trust, Birmingham, United Kingdom

## Abstract

**Aims:**

Schizophrenia affects an estimated 24 million people worldwide, with around one third experiencing inadequate response to first-line antipsychotics. Clozapine is the most effective treatment for this group but is reserved as a last-line option due to serious adverse effects and intensive monitoring requirements. Its metabolism varies widely due to age, smoking, genetics and ethnicity. Significant risks include neutropenia, myocarditis, metabolic syndrome. We present the case of a patient with clozapine-induced dysarthria, an uncommon but debilitating side effect. The associated reduced speech intelligibility and difficulties in communication can cause considerable distress for patients who are already coping with the challenges of a psychiatric disorder, and increases the chances of poor adherence to the medication.

**Methods:**

Patient A is a male in his 40s who developed schizophrenia during early adulthood. He was diagnosed with treatment-resistant schizophrenia; Clozapine trials were complicated by dose-related adverse effects, most notably that of sudden onset dysarthria for which no other cause was identified. Rechallenge was further complicated by adverse cardiovascular effects. Clozapine plasma levels were found to be consistently raised despite relatively low doses of Clozapine, suggesting that the patient was a poor metaboliser of the drug. Progressive dose reductions improved speech while maintaining psychiatric stability. The dysarthria subsequently improved on low-dose clozapine with adjunctive sodium valproate.

**Results:**

The presented case demonstrates a clear chronology of the development of dysarthria at sub-therapeutic doses of Clozapine, and its subsequent resolution with reduction of the dose. Existing literature describes similar clozapine-associated speech dysfluency, more commonly stuttering, often showing a dose-response relationship. Some cases link symptoms to orofacial dyskinesia, prior neurological vulnerability, or possible micro-seizure activity, even with normal EEG findings. Proposed mechanisms include reduced seizure threshold via dopaminergic selectivity, GABAergic inhibition, glutamatergic enhancement, and anticholinergic effects. Prophylactic sodium valproate may improve the dysarthria, and allow continuation of clozapine in selected high-risk patients.

**Conclusion:**

This case emphasises dysarthria as a dose-related adverse effect of clozapine, particularly in patients with unexpectedly high plasma levels. It highlights the importance of close therapeutic drug monitoring during titration and consideration of individual metabolic factors such as ethnicity and comorbidities. When speech dysfluency emerges, clinicians should assess potential risk of clozapine-associated seizures and consider EEG investigation. Anecdotal evidence suggests sodium valproate may alleviate speech disturbance even without overt seizure activity. Further research is needed to clarify the mechanisms underlying clozapine-related speech dysfluency and to inform strategies that balance efficacy and tolerability in treatment-resistant schizophrenia.

